# Proteoform Analysis of the Human Olfactory System: A Window into Neurodegenerative Diseases

**DOI:** 10.3390/proteomes12010009

**Published:** 2024-03-21

**Authors:** Eqrem Rusi, Fiorenza Pennacchia, Wael Abu Ruqa, Giuseppina Talarico, Giuseppe Bruno, Antonio Minni, Christian Barbato

**Affiliations:** 1 Department of Human Neuroscience, Sapienza University of Rome, 00185 Rome, Italy; rusi.1786405@studenti.uniroma1.it (E.R.); giuseppina.talarico@uniroma1.it (G.T.); giuseppe.bruno@uniroma1.it (G.B.); 2 Department of Sense Organs DOS, Sapienza University of Rome, Viale del Policlinico 155, 00161 Roma, Italy; pennacchia.1748833@studenti.uniroma1.it (F.P.); antonio.minni@uniroma1.it (A.M.); 3 Division of Otolaryngology-Head and Neck Surgery, Ospedale San Camillo de Lellis, ASL-Rieti-Sapienza University, Viale Kennedy, 02100 Rieti, Italy; wael_97@hotmail.it; 4 Institute of Biochemistry and Cell Biology (IBBC), National Research Council (CNR), Sapienza University of Rome, Viale del Policlinico 155, 00161 Roma, Italy

**Keywords:** olfactory proteome, olfactory dysfunctions, Alzheimer disease, neurodegenerative disease, SARS-CoV-2

## Abstract

**Background**: Very little is known about the proteome of the human olfactory system and how diseases associated with olfactory dysfunctions can affect it. With this review, we try to summarize the existing literature on the use of this technique for a better understanding of the neurodegenerative disease process. **Methods**: We used the PubMed database and found different articles which were then selected independently by three authors. **Results**: We found 157 articles, of which, after careful selection, only 30 were analyzed in this review. We presented all the associations identified between the protein/pathway alterations neurodegenerative diseases and SARS-CoV-2 infection. **Conclusions**: We think that the proteome of the olfactory system through blood, saliva, and mucus analysis could be a new way to better understand, diagnose, and finally treat neurodegenerative diseases.

## 1. Introduction

The human olfactory system is responsible for the detection, transmission, and identification of odorous stimuli [1]. To encode the odors, the olfactory pathways are connected by several circuits, comprising approximately 400 peripheral olfactory receptors, about 10^7^ olfactory sensory neurons (OSNs), and central structures, where olfactory information is recognized and stored [2]. The OSNs expressing single types of olfactory receptors are bipolar neurons located in the context of the mucosa and they represent the first neurons of the pathway. Their dendritic extensions are effective in transforming molecular information into electrical stimuli. Together, these extensions give rise to the olfactory nerve, which finally reaches the olfactory bulb (OB) [3].

OB are small telencephalic structures composed of five layers arranged around a central core of white matter, from which the olfactory tract originates [3]. The OB contains the second neurons of the pathway, the projection neurons. The synapse between the first neurons and the second neurons of the pathway occurs at the level of the glomeruli, a spherical structure contained in the OB [3]. The olfactory tract then leads to the anterior perforated substance and, in their course, the axons of the olfactory tract emit collaterals which synapse with the neuronal cells of the anterior olfactory nucleus. From this relay, a projection also reaches the contralateral OB through the medial olfactory stria and the anterior commissure [3]. Once it reaches the anterior perforated substance, the olfactory tract expands into the olfactory trigon, from where the olfactory striae originate lateral olfactory stria, intermediate olfactory stria, medial olfactory stria [3].

The fibers that originate from the OB and run through the olfactory tract without thalamic retransmission directly reach the olfactory cortex of the cerebral hemisphere, where the largest part belongs to the piriformis lobe in the temporal area, the most phylogenetically ancient areas composed of paleocortex [3].

The entorhinal cortex corresponds to Brodmann’s area 28. The lateral areas of the cortex receive afferents directly from the OB, the pre-piriformis cortex, and the pre-amygdaloid. Finally, its connections aim extensively at the limbic system, the area linked to synaptic transmission, cognition, and memory. Although the amygdala and hypothalamus are not cortical areas, they still receive direct bulbar projections [3].

In the description of the neuroanatomy of the olfactory system, it is important to mention some important and new concepts. The outermost layer of the olfactory bulb consists of compacted axons of the olfactory nerve whose axons are dispersed before passing through the cribriform plate. The synaptic glomeruli of the olfactory bulb are present only on the ventral surface facing the cribriform plate and likely correspond to the peripheral ganglia of other special sensory cranial nerves but are otherwise lacking in the olfactory nerve and have led some authors to suggest that the olfactory is not a true cranial nerve [4]. The olfactory is the only special sensory system to not project primarily to the thalamus because the OB and tract incorporate their intrinsic thalamic equivalent in the form of the granule cell core of the bulb that extends into the olfactory tract; these granule cells have many dendro-dendritic synapses between them, like the thalamus. These features are best understood in the context of the embryonic and fetal development of the olfactory system [3,4].

As background for known essential functional factors to sensitive odor identification and discrimination, it is important to consider olfactory receptors and olfactory bulb dopaminergic cells. Primarily, the genetic ablation of dorsal olfactory receptors induced severely impaired fox-characteristic odor recognition and enantiomer odor discrimination with maintained high sensitivities or partially impaired sensitivities in odor detections [5,6]. On the other hand, the rostral migratory stream of the newborn dopaminergic neuroblasts from the subventricular zone of the ventral telencephalon to the OB [7] and the association between severely impaired odor detection and reduction in dopaminergic small cells enhanced the mobilization of subventricular zone progenitor cells in the OB of genetic Parkinson’s disease model mice [8].

Despite its importance at diagnosis, actual patients’ pathogenesis-associated proteomic profiles of olfactory receptors or subsequent pathways in mammals remain unknown.

The sense of smell is susceptible to several factors. Most of them result from diseases of the nasal or paranasal sinuses, head trauma, and upper respiratory infections that harm the olfactory neuroepithelium. The alteration of the sense of smell in pathological terms can also follow the effect of age, smoke, toxic substances, psychiatric disorders, intracranial lesions, epilepsy, and neurodegenerative diseases (NDs), such as Alzheimer’s, Parkinson’s, and Huntington’s disease (AD, PD, and HD) [9,10]. Anosmia is one of the first symptoms of these NDs [1].

AD results from the incorrect proteolysis of the amyloid precursor protein (APP), developed in about 10–20 years, which causes the extracellular accumulation of aggregates of amyloid β (Aβ) substance, the amyloid plaques, and the interneural accumulation of neurofibrillary tangles of the hyperphosphorylated tau protein in encephalic areas such as the hippocampus limbic system, entorhinal and temporal cortex. After years, the first symptoms appear, including cognitive decline up to the state of dementia [10].

Moreover, one of the most frequent causes of loss of smell is the infectious damage caused by the SARS-CoV-2 viral agent, the etiological cause of COVID-19 disease. A growing number of evidence shows that olfactory damage (OD) may be caused by both mechanical damage, such as the inflammatory obstruction of the roof of the nasal cavity at the cribriform plate, and by structural damage, such as the neuronal insults on the different level of olfactory system, according to cellular mechanisms not clarified [11,12]. During the first COVID-19 outbreak, when it was considered a pandemic, OD was one of the main symptoms frequently described by patients [13,14].

In the last few years, proteomics has evolved into a comprehensive and extensive approach for examining and measuring the activity of specific systems. Proteome’s study associated with olfaction in diverse biological contexts was developed. These contexts can encompass neurodegeneration, aging, olfactory learning, and other related conditions [2].

Given that recognizing distinct proteomes associated with normal physiological processes is crucial for understanding olfactory mechanisms, various proteomic characterizations of olfactory proteins have been documented. Despite extensive endeavors to characterize the proteins found in the olfactory system of murine models, the description of proteins in the human OB remains notably limited [2].

Usually, considering the intricate cellular composition and diversity of proteins within the OB, the application of high-resolution mass spectrometry (MS) for proteome-wide analysis, integrated with the bioinformatic analysis of proteins, has emerged as an appealing technology [15,16].

This approach proves valuable in characterizing and quantifying the OB proteome across various biological scenarios [17].

This review aims to collect a description of a human olfactory proteomic profile which can assist in identifying valuable biomarkers for the early diagnosis and prognosis of various diseases which include olfactory loss, such as NDs and post-COVID-19 olfactory disorders. Indeed, the study of proteomics, integrated with genetics, would help us understand the biochemical basis of the olfactory pathway and the etiopathogenesis of disorders linked to smell.

## 2. Materials and Methods

**Figure 1:**
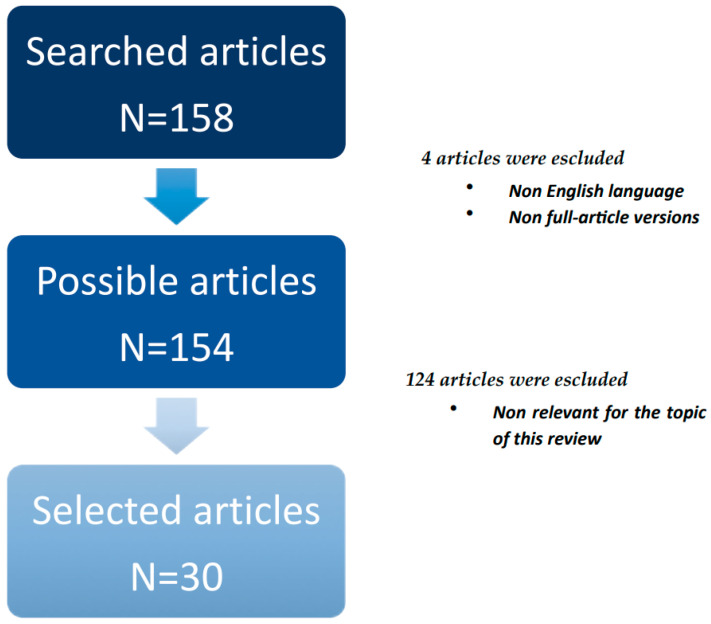
Flow chart showing the process of article selection.

We used the PubMed database to search through the literature. For this search, the terms used were: (proteome* OR proteomics) and (human smell) or (olfactory bulbs) (olfactory nerve) or (olfactory cortex). As a result, we identified 158 possible articles (Figure 1). We then excluded articles published in a language different from English and those articles in which the full text was not available. In total, 154 records remained. Afterward, the titles and abstracts were initially screened and selected by three independent reviewers (F.P., W.A.R., and E.R.) based on their relevance to the review topic. As a result, 124 studies were excluded. The remaining 30 studies were then analyzed and discussed in this review.

## 3. Results

### 3.1. Olfactory Proteomic

“Omic” analysis of olfactory proteins has the potential to be a dependable means for validating biomarkers related to OD due to ND, post-viral infections, and idiopathic causes. Protein extracts from the OB can be an important source for identifying the physiological and pathological changes of the organism [2].

The presented report provides a limited perspective regarding the mentioned aspects but underscores the potential for further exploration of human OB proteomics. Starting from the consideration that the OB’s anatomy is well-defined region with distinct spatial patterns of gene expression, the advancement of precise isolation and purification protocols for individual cell types within the OB, coupled with recent advancements in shotgun proteomic methods, could enable the exploration of transcriptome and proteome profiles for each OB cell population separately. This approach would enhance our molecular understanding of the OB [2].

Numerous proteins released from the olfactory pathway which contain several neurons offer a promising avenue for profiling in the context of olfactory disorders. Unfortunately, the study of human olfactory proteomics is an invasive method, as the tissue of the olfactory tract comes from autopsy specimens. Most of the studies in the literature have been conducted on murine models or in human post-mortem [1].

From a physiological standpoint, the predominant proteins found through the technique of high-resolution MS for proteome-wide analysis in human OB include about a thousand macromolecules. Given that in most cases OD is in the advanced stages of pathologies like neurological diseases and viral infections, conducting an early analysis of olfactory tract protein content could prove to be a beneficial strategy in unveiling the presence of the principal disease [18].

Dammalli et al., in their first human olfactory tract proteome study, discovered a total of 6055 proteins and subjected them to bioinformatics analysis and contextualization to discern the associated biological processes and molecular functions [18]. The identified proteins were associated with processes and functions related to olfactory perception, cell-to-cell adhesion, cellular and G protein-coupled receptor activity, axonal growth, and transportation. Notably, 83 proteins were restricted to the olfactory tract, while 4 seven-transmembrane proteins, and 14 protein kinases were identified in this study [19].

In their subsequent work, they also detected 7750 non-redundant proteins contained in the human OB, and using bioinformatics analysis, they demonstrated their role in biological processes related to signal transduction, metabolism, transport, and olfaction [20]. We suggest keeping in mind that the functioning of the olfactory tract is susceptible to the action of age, smoke, toxic substances, psychiatric disorders, intracranial lesions, epilepsy, and neurodegenerative diseases. This aspect is also explored in proteomics applications. For example, Hao-Long Zeng and colleagues demonstrated that changes in the proteome induced by odor deprivation and stimulation distinctly illustrate substantial metabolic shifts and modifications in synaptic transmission. This quantitative systems biology investigation brings about an enhanced comprehension of OB plasticity development triggered by either odor deprivation or stimulation. Moreover, it offers numerous new insights for conducting functional studies related to the OB [21]. An important prospect of the olfactory metabolism proteomic approach is given by Mercedes Lachén-Montes et al.’s work, trying to define the dark proteome, made by protein components of human chromosomes with unknown function [22].

The dark C1orf128 (PITHD1) protein, also known as uPE1 (C-terminal proteasome-interacting domain of thioredoxin-like) domain-containing protein 1, previously identified as a component of the human central proteome, plays a role in various biological processes such as megakaryopoiesis, ovarian carcinoma, and male fertilization. However, the specific functional features associated with the PITHD1 gene remain poorly understood. The PITHD1 gene, positioned on chromosome 1 (1p36.11), is a highly conserved protein-coding gene [22]. Through the utilization of diverse bioinformatic tools, several key observations have been made: (1) PITHD1 tends to localize within the nucleus, (2) it undergoes eight phosphorylations, five ubiquitinations, three O-GalNAc modifications, and one methylation, (3) and analyzing the co-regulation map of the human proteome, it has been noted that PITHD1 tends to co-regulate with mitochondrial protein components, particularly those engaged in nucleotide metabolism, mitochondrial transport, and autophagy [22]. PITHD1 has been identified in the human olfactory neuroepithelium (ON) [23], a tissue that undergoes continual neurogenesis and because of lesions, regenerates to maintain sensory function [24]. The ON is frequently used as a surrogate for central nervous system (CNS) function in research [25]. Notably, a subset of proteins co-regulated with PITHD1 has been associated with neurological disorders such as AD and PD [26]. Furthermore, a portion of the deregulated proteome in the OB during the progression of AD and PD corresponds to proteins co-regulated with PITHD1. These findings suggest a potential connection between PITHD1, mitochondrial function, and the pathogenesis of neurological disorders, particularly in the context of AD and PD progression [27].

This analysis was conducted in human olfactory neuroepithelial cells, focusing on the PITHD1 protein [22]. To characterize the functional role in these cells, a combination of antibody arrays was employed. This approach aimed to probe and delineate changes in the activation patterns of kinases and transcription factors. The use of antibody arrays allowed for a comprehensive examination of the functional properties of PITHD1 in the context of human ON cells, shedding light on its potential implications in various cellular processes. Notably, PITHD1 was found to stimulate the production and secretion of multitasking cytokines, including IL-6 and IL-8, as well as chemokines in ON cells [28]. This indicates that the introduction of exogenous PITHD1 induces a pro-inflammatory phenotype in ON cells. Of particular significance, IL-6, which is highly sensitive to olfactory sensory neuron (OSN) injuries, is among the cytokines influenced by PITHD1 [29]. The research indicates a potential association between PITHD1 and age-related olfactory impairment, suggesting that PITHD1 might play a role in modifying olfactory function, possibly by triggering a pro-inflammatory response in ON cells. In individuals with AD, a notable degeneration of axons has been identified in the olfactory tract, and this degeneration corresponds with the onset of dementia. Additionally, structural changes and atrophy in the olfactory tract have been observed in individuals with PD compared to controls. Interestingly, the expression of PITHD1 in the olfactory tract was significantly higher in individuals with AD compared to controls. Furthermore, no significant changes in PITHD1 expression were observed in the olfactory tracts derived from individuals with PD. This suggests that elevated expression of PITHD1 in the olfactory tract may be specifically associated with Alzheimer’s disease and not Parkinson’s disease. The findings further support the idea that PITHD1 could be involved in age-related olfactory dysfunction, particularly in the context of neurodegenerative conditions like Alzheimer’s disease [30].

### 3.2. Proteomic Studies on SARS-CoV-2 Associated Olfactory Dysfunctions

Loss of smell is one of the first signals of SARS-CoV-2 viral infection, the etiological cause of COVID-19 disease. The onset of SARS-CoV-2 infection initiates with the attachment of the viral glycoprotein spike S protein to angiotensin-converting enzyme 2 (ACE2), a receptor belonging to the human olfactory epithelium (OE). It is widely recognized that viral proteomes consist of various arrangements of protein–protein interactions (PPIs) to fulfill a wide range of essential functions throughout the viral life cycle [31].

SARS-CoV-2 is not the only virus to cause olfactory dysfunctions. The Zika virus (ZIKV) is a recently identified arbovirus belonging to the flavivirus genus and is linked to congenital Zika syndrome (CZS) in infants. The susceptibility and biological responses of the olfactory system to ZIKV infection has been analyzed using both mouse models and human olfactory organoids derived from the olfactory mucosa of patients. The study’s findings reveal that neonatal mice infected with ZIKV experience temporary olfactory dysfunction that persists until puberty. This indicates that the olfactory system is a significant target for ZIKV infection, and it suggests that post-viral olfactory dysfunction may be overlooked in patients with congenital Zika syndrome [32].

Concerning SARS-CoV-2, several studies have been conducted on animal models, mainly in mice. A study showed that SARS-CoV-2 affects the olfactory epithelium (OE) and induces temporary olfactory dysfunction in mice expressing human ACE2 (hACE2). Therefore, a mouse model with humanized ACE2 (hACE2) has been created, rendering it susceptible to SARS-CoV-2 infection. As anticipated, elevated levels of SARS-CoV-2 RNA were identified in the nasal respiratory epithelium (RE), trachea, and lungs at both 2 and 4 days post-infection (dpi). The highest level of viral RNA (2.36 × 10^11^ RNA copies/mouse) was observed in the lungs at 2 dpi. Additionally, substantial expression of the viral nucleocapsid (N) protein was observed in the lungs of hACE2 mice infected with SARS-CoV-2 at both 2 and 4 days dpi, whereas no such expression was detected in the control animals [33].

In line with this, to enhance serology tests for quick COVID-19 screening, there have been advancements in the development of antibody microarrays capable of detecting SARS-CoV-2 antigens. These microarrays aim to chart the humoral response in individuals with COVID-19. These findings validate the clinical data that demonstrate an innate immune response and the subsequent occurrence of a cytokine storm during SARS-CoV-2 infection, suggesting that the search for the pathway of human-released proteins may be a biomarker to understand the etiopathogenesis of this disorder. From these investigations, the sensitivity and efficiency of specific MS techniques can enhance current clinical genetic studies relying on polymerase chain reaction (PCR) and they can be applied also to human molecular patterns. This technological advancement stands as a crucial pillar to be considered for future assistance in addressing the global spread of the novel coronavirus responsible for COVID-19 [34].

In a study conducted on rhesus monkeys, it was shown that SARS-CoV-2 enters the central nervous system (CNS) primarily through the olfactory bulb (OB). Despite suggestions that the olfactory nerve may not be a probable route for brain infection in COVID-19 patients, various reports have indicated the presence of SARS-CoV-2, including its mRNA/protein levels or viral particles, in multiple brain regions, including the OB, from individuals with COVID-19 [35].

While the characterization of the SARS-CoV-2 genome and proteome offers valuable insights into the virus’s structure and protein interactions, gaining an understanding of how the infection disrupts metabolic homeostasis in COVID-19 patients could offer valuable insights for developing innovative diagnostic and therapeutic interventions [35].

### 3.3. Proteomic Studies on Neurodegenerative Diseases Associated with Olfactory Dysfunction

Not only COVID-19, but also other diseases are characterized by olfactory dysfunction. It is thought to be an early marker of neurodegenerative diseases, being diseases primarily characterized by neuron loss [1].

However, the mechanism is not completely clear but is thought to be caused by the structural modifications in the olfactory bulb as the result of protein aggregates accumulation. The growing prevalence and disability of these pathologies, for which in many cases we do not have a disease-modifying treatment or early diagnostic tests, pushes research into finding new ways to study these conditions. One of them is the base of the proteomic approach [36].

In this part of the review, we will first present studies that generally associate olfactory proteomic with neurodegenerative diseases, and then we will discuss separately the most important diseases, which afterward will be summarized.

The olfactory bulb is in connection with many cortical areas. For example, the entorhinal and frontal cortex are crucial in NDs. The entorhinal cortex is associated with the “working memory”. It is one of the earliest areas involved in AD, and it is one of the first brain areas where τ protein aggregations are found. The atrophy of this area is surely due to neural loss, but also microglial and/or astroglia involvement, as suggested by Astillero-Lopez et al. [37]. From the proteomic analysis, it emerged that these processes are correlated with the upregulation of some proteins (S100A6, PPP1R1B, BAG3, and PRDX6) and the downregulation of others (GSK3B, SYN1, DLG4, and RAB3A). These clusters were related to synaptic, neuro-inflammatory, and oxidative stress processes [37]. Also, Salimi et al. suggest that this cortex is functionally associated with the OB, and proved in animal studies that the olfactory bulb modulates entorhinal cortex oscillations during spatial working memory [38]. Proteomic analysis of the entorhinal cortex can help identify new ways of understanding NDs. For example, the dysregulation of ion transport in the entorhinal cortex of AD patients is thought to be a signaling pathway that initiates pathology [39].

Animal studies showed the functional connection between the OB and the frontal cortex. Rodent bulbectomy caused significant biochemical and structural alterations that could explain the frontal cortical abnormalities observed like depression [40]. This association could also explain the behavioral symptoms that characterize many NDs.

Cartas-Cujedo, in 2021, explored the olfactory protein homeostasis in neurological patients affected by different spectrums of smell dysfunction. They used a proteoform approach and OB proteomics datasets derived from subjects with AD, PD, mixed dementia, dementia with Lewy bodies, frontotemporal lobar degeneration (FTLD-TDP43), progressive supranuclear palsy (PSP), and amyotrophic lateral sclerosis (ALS), were compared and integrated. They focused on the OB because it is the first olfactory brain area resident of neuropathological and molecular changes. They identified that the purine nucleoside phosphorylase (PNP) was the only over-expressed protein in AD, PD, MixD, and ALS. Thirteen OB proteins were differentially regulated in at least three neurological disorders, of which four of them (NCAM2, LY6H, COL6A3, and PRDX6) presented a homogeneous OB profile across diseases [41]. In addition, the epidermal growth factor receptor (EGFR) expression was significantly increased in the OB of AD and mixed dementia subjects [42].

Cartas-Cujedo and colleagues used olfactory proteomics to search for novel sex-biased physiopathological mechanisms associated with olfactory dysfunction. The scientific basis of this research is found in the sex differences in terms of smell and olfactory structures as well as in the manifestation of both neurological syndromes. They studied the olfactory tracts of PD and AD patients. Overall, they found that the tangled SIRT (SIRT1-5) profile observed across the olfactory pathway-associated brain regions in AD and PD indicates differential NAD (+)-dependent deacetylase mechanisms between women and men [43].

#### 3.3.1. Alzheimer’s Disease

Knowing the growing prevalence of AD, many studies were completed to understand the molecular changes in the brains of AD patients. Another proof of the connection between olfactory dysfunction and AD is found in the study of Bubak et al., where they analyzed the proteome of OB and OT of familial AD patients. In this study, it is interesting that in all members of the family in this study, signs of viral infection and inflammation of the OB and OTs were found. Furthermore, they conclude that the viruses cause alterations of the olfactive pathways, and as a result, a dysregulation of the OT, accelerating cognitive decline. These results imply that a characteristic of FAD is the impaired communication between OB and hippocampus [44].

Still, it remains unclear which biochemical alterations cause the olfactory dysfunction in AD. Some authors propose SREBF1 and NFMUE1 (involving genes with 3′UTR containing motif CGGCCATCT) as transcription factors with a specific role of mediators relevant in AD olfactory neurodegeneration [41].

The OB proteome profile of AD patients changes during the evolution of the disease, as stated by Braak stages [45]. In advanced AD stages, a significant increment of STAT3 was observed. The progression of MixD was not differentially modulated across AD staging from a proteoform point of view. Selected altered proteins are the cAMP-responsive element binding protein 1 (CREB1) and the AP-1 transcription factor subunit (c-Jun) [46]. In a study conducted by Lachén-Montes et al., they analyzed the OB-proteome to identify a stage-dependent synaptic proteostasis impairment during AD evolution [46]. They found progressive modulation of τ and amyloid precursor protein (APP) interactomes, early disruption of upstream and downstream p38 MAPK pathway, and subsequent impairment of protein kinase C (PKC) signaling axis in the OB from AD subjects. Moreover, a mitochondrial imbalance was evidenced by a depletion of prohibitin-2 (Phb2) levels and a specific decrease in the phosphorylated isoforms of Phb1 in intermediate and advanced AD stages. Phb2 showed a specific up-regulation in mixed dementia, while Phb1 isoforms were down-regulated in FTLD and there were no differences in PSP. The proteoform analysis evidenced that the Phb complex can be a differential driver of olfactory neurodegeneration [17].

Another interesting fact is the sex difference found in the proteomic studies of the olfactory tract in AD patients, where differentially expressed proteins were observed in AD women and men, with 327 and 151 proteins, respectively, while only 35 shared proteins. Perhaps these differences are the expression of the sex effect in the ability to sense smells and/or the clinical manifestation differences between male and female sex [43].

As was mentioned before, nearly all proteomic studies were conducted in murine models or human post-mortem, because of how invasive these studies are. In recent years, new studies have emerged, using the proteomic analysis method for blood, saliva, and mucus analysis; Yoo et al. searched soluble Aβ on nasal discharge of patients with mild and moderate cognitive decline and in a control group [47]. Higher levels of Aβ oligomers were found in probable AD patients with lower MMSE and a higher Clinical Dementia Rating and Global Deterioration Scale. Also, mild and moderate subjects can be distinguished because of the increased concentration of Aβ56 in the mild group and 15-mer AβO for the moderate group [48].

#### 3.3.2. Frontotemporal Dementias

Mild olfactory dysfunction has been seen also in frontotemporal dementias (FTD). Lachén-Montes and colleagues in 2019 study applied quantitative proteomics to analyze pathological effects on the olfactory bulb in subjects with supranuclear palsy (PSP) and frontotemporal lobar degeneration (FTLD-TDP43). Concerning the elderly non-FTD group, proteomic analysis of the OB showed that these two clinically similar FTD disorders have a different neuropathological hallmark. Both presented alterations of the following pathways: mitogen-activated protein kinases (MAPKs), calcium/calmodulin-dependent protein kinase II (CAMKII), and PKC. In the PSP patients alterations of the mitogen-activated protein kinase kinase 4 (SEK1/MKK4)/stress-activated protein kinase (SAPK) pathways were found. In the FTLD-TDP43, there was a specific phosphoinositide-dependent protein kinase 1 (PDK1) inactivation [49]. Another finding on FTLD-TDP43 patients is the specific enrichment of proteins regulated by NMYC [41].

#### 3.3.3. Lewy-Type α-Synucleinopathies

Other neurodegenerative pathologies characterized by olfactory dysfunction are the Lewy-type α-synucleinopathies. The most famous one is certainly PD. In an animal study conducted in mouse models expressing human α-syn, it was demonstrated that the accumulation of this protein was associated with a reduction in OB neurogenesis [50].

In a human study, researchers used mass spectrometry–based quantitative proteomics in the post-mortem OB of Parkinsonian patients across limbic, early neocortical, and neocortical LTS stages of the disease. The proteostasis impairment was observed, identifying 268 differentially expressed proteins between controls and PD phenotypes. Also, network-driven proteomics revealed a modulation in ERK1/2, MKK3/6, and PDK1/PKC signaling axes [51]. At the transcriptional level, bioinformatics analysis predicted HDAC1, CIITA, MYC, CDC5L, USF, and PTF1β as potential regulators of the OB-modulated proteome in PD cases [41].

Proteomic studies evaluating sex differences in the PD identified 198 differentially expressed proteins in PD women, whereas 95 differentially expressed proteins were detected in PD men with 20 in common [43].

A proteoform analysis in DLB showed that the protein interaction networks indicated an imbalance in translation and the synaptic vesicle cycle, which were accompanied by alterations in AKT/MAPK/SEK1/p38 MAPK signaling pathways [52].

#### 3.3.4. Mixed Dementia

Mixed dementia (MixD) is a form of dementia defined by the presence of abnormal protein deposits, characteristic of Alzheimer’s disease (AD) with the coexistence of vascular disease. Little is known about the olfactory dysfunction in MixD. For this reason, Lachén-Montes and colleagues studied the PB proteome of six MixD subjects concerning seven normal controls. Studying the involved pathways and the differences with AD, they propose serum low-density lipoprotein receptor-related protein 1 (LRP1), also known as alpha-2-macroglobulin receptor (A2MR) or CD91, involved in various biological processes via interactions with >100 different extracellular ligands and several intracellular proteins [53], as a candidate marker to differentiate AD and MixD [54].

In MixD the proteomic analysis revealed that Nuclear Factor I/C was the only transcription factor potentially involved in OB proteoform imbalance in this neurological disease. Enrichment analysis pointed out that proteoglycans, ADP-ribosylation factor 3 (ARF3) pathway, NOVA-regulated synaptic proteins, NOTCH3 signaling, synthesis of GPI-anchored proteins, glutathione conjugation and phosphatidylinositol signaling were part of the pathways selectively enhanced in MixD cases [41].

#### 3.3.5. Amyotrophic Lateral Sclerosis

In ALS patients with non-motor symptoms, the olfactory dysfunction is reported. A proteoform study using a quantitative proteotyping approach of the OB and OT in ASL subjects evidenced that the over-production of the olfactory marker protein (OMP) points out an imbalance in the olfactory signal transduction in ALS. Accompanying the specific over-expression of the glial fibrillary acidic protein and Bcl-xL in OT, a puzzled disruption of signaling routes was evidenced across the OB–OT axis in ALS [55]. Furthermore, deregulated proteins were mapped in cell–cell contact zones and ciliary basal bodies, potentially regulated by STAT3 and involved in IL-18 signaling and complement systems, RAS processing, and amoebiasis [41].

**Figure 2:**
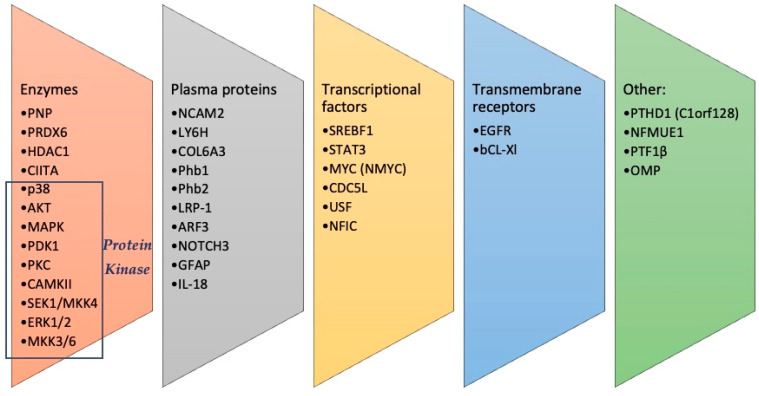
A summary of the identified molecules, divided into different categories.

In Table 1 and Figure 2, we present a synthesis of proteomic studies in NDs associated with olfactory disease. Table 1 shows the molecules/pathways involved in the different neurodegenerative syndromes [41], while in Figure 2, the identified molecules are divided into five categories.

## 4. Discussion

Due to the complexity of the human brain, the characterization of proteome profiles within specific areas, brain structures, and biofluids, is essential to understanding the biochemical basis of structural specialization and alterations associated with diseases involving the sense of smell [12].

In the coming years, the utilization of anatomical delineation and sample isolation through techniques such as dissection or laser microdissection, coupled with advanced proteomic strategies, is poised to advance our understanding of cell type- and olfactory region-specific proteomes expressed in the human olfactory system. However, despite the awareness that approximately 4% of the human genome may play a role in olfaction, olfactory neuroproteomics is currently insufficient for comprehensively decoding the smell function at the proteomic level.

Furthermore, considering the dynamic nature of olfactory transcriptomes and proteomes during neurological development, further proteomic investigations involving nasal biopsies and post-mortem brains from donors without obvious relevant clinical features, by gender, age, and ethnic origins, will be essential to build a comprehensive proteomic profile of human olfaction.

An important boost for olfactory research has undoubtedly been generated by the COVID-19 pandemic because one of the most evident consequences both in the acute and long-term phases of the infection has been characterized by alterations of smell with the involvement of the nasal epithelium [56].

Recently, neurons were infected with defective SARS-CoV-2 or treated with preconditioned media from SARS-CoV-2 infected lung cells, to evaluate the proteomic expression profile, evidencing an involvement of mitochondrial proteins potentially involved with the neurological consequence of viral infection [57].

In this regard, it could be interesting to analyze these neuroproteomic profiles with those obtained from the analysis of smell reported above and evaluate potential overlaps or interactions concerning olfactory stimuli.

On the other hand, there is a compelling need to develop non-invasive methods that can measure the sense of smell in human diseases. Recently, a diagnostic method for olfaction was developed using an optical probe of voltage-gated sodium channel 1.7 to measure and obtain a non-invasively image expression of NaV1.7. This fluorescent imaging agent represents an advanced non-invasive diagnostic method to evaluate human smell, connecting the proteomics results of NaV1.7 impairment at OB and olfactory epithelium levels [58].

Interestingly, another non-invasive test for an accurate diagnosis was the electrobulbogram, a functional measurement of the OB, which is one of the first processing areas of the olfactory CNS, which undergoes degeneration in Parkinson’s patients also several years before the most serious manifestations [58].

The spectrogram of the electrobulbogram showed a specific profile not only following olfactory stimuli, but also related to the pharmacological treatment and clinical characteristics of identifying odors of Parkinson’s patients. This non-invasive examination could open new approaches to link the differentiating patterns of odor-related synchronization in the frequency bands and proteoform analysis.

To conclude this point, another non-invasive examination for accurate diagnosis of olfactory diseases is represented by nasal swabs.

Nasal swabs are a non-invasive explorative method able to individuate diseases by collecting samples from the nasal cavity. Nasal swabs are being used to explore neurodegenerative diseases using the seed amplification assay (SAA) of pathogenic misfolded proteins, such as prion, α-synuclein, and tau. The misfolded proteins can serve as a template for proteins of the same type to misfold. The SAA of nasal swab extracts is a technique that starts from very low amounts of misfolded protein aggregates in biofluids to detect the seeding activity of neuropathological proteins [59]. The future of olfactory proteomics depends on new methodological and sample processing capabilities and integration with other “omics” approaches, such as the electronic nose. This approach is crucial to unveil the diversity of proteins involved in olfaction and identify molecular determinants in the early development of some NDs characterized by cognitive deficits, such as tauopathies, tuberous sclerosis complex, hemimegaloencephaly and cortical dysplasia focal type II, characterized by phosphorylated tau, such as Down syndrome not in childhood but in early adult life, for which olfactory proteins have not been systematically studied.

## 5. Conclusions

The study of the olfactory system through proteomic analysis is a new tool for understanding the physiology of the human olfactory system, consequently obtaining information on the progress of diseases involving the sense of smell. In this short review, we have introduced the importance of proteomics and its applications, through the identification of proteoform peptides with pathological significance, even if these studies were executed mostly in human post-mortem tissues. Therefore, all the results showed a faint clinical impact. Due to the incomplete knowledge of the biological mechanisms, we are far from identifying possible target therapies. Nonetheless, it could be simpler to use this knowledge for diagnostic purposes. It would be a huge step forward if we could validate non-invasive analyses that could help us diagnose these diseases in an early phase or even in a preclinical one, becoming a screening instrument for neurodegenerative conditions. The blood, saliva, and mucus analysis are an essential source of biological samples useful to investigate the proteoform profile of the olfactory system. In our opinion, the integration of proteoform-focused proteomic studies [60], system biology, and artificial intelligence considering the olfactory system based on biofluids as nasal secretions, can be a door to better understanding the whole nervous system and its degenerative pathologies. Finally, these biofluids could be the way to study the brain proteomic in vivo and not by autopsy.

## Figures and Tables

**Table 1:** A summary of the results of the analyzed studies. In BOLD, the proteins/pathways involved in AD, PD, MixD, ASL. Upregulated or downregulated proteins were indicated by bold letters: bold letters upregulated; bold letter underlined downregulated, and bold italic partially downregulated. In RED, exclusively AD and MixD. In GREEN, exclusively in AD, FTD and PD/LBD. In PURPLE, AD and LBD.

Neurodegenerative Diseases	Single Proteins/Pathway Alterations
Alzheimer disease	**PNP**, **NCAM2, *LY6H*, *COL6A3*, *PRDX6***, EGFR, SREBF1, NFMUE1, STAT3, p38, MAPK, PDK1, PKC, Phb2
Fronto-Temporal Dementia	MAPK, CAMKII, PKC
PSP	SEK1/MKK4, SAPK
FTLD-TDP43	PDK1, NMYC, Phb1
Lewy-type α-synucleinopathies	
PD	**PNP**, **NCAM2, LY6H, COL6A3, PRDX6**, ERK1/2, MKK3/6, PDK1/PKC, HDAC1, CIITA, MYC, CDC5L, USF, PTF1β
LBD	AKT/MAPK/SEK1/p38 MAPK
Mixed dementia	**PNP**, **NCAM2, LY6H, COL6A3, PRDX6**, EGFR, Phb2, LRP-1, NFIC, ARF3, NOTCH3
Amyotrophic lateral sclerosis	**PNP**, ***NCAM2*, *LY6H*, *COL6A3*, *PRDX6***, OMP, GFAP, Bcl-xL, IL-18
